# Use of dolutegravir in two INI-experienced patients with multiclass resistance resulted in excellent virological and immunological responses

**DOI:** 10.7448/IAS.17.4.19755

**Published:** 2014-11-02

**Authors:** Laura Marije Hofstra, Monique Nijhuis, Tania Mudrikova, Axel Fun, Pauline Schipper, Margriet Schneider, Annemarie Wensing

**Affiliations:** 1 Medical Microbiology, University Medical Center Utrecht, Utrecht, Netherlands; 2 Infectious Diseases and Internal Medicine, University Medical Center Utrecht, Utrecht, Netherlands

## Abstract

**Introduction:**

Dolutegravir is a second generation integrase inhibitor with a proposed high genetic barrier to resistance. However, in clinical trials, decreased virological response was seen in a subset of patients with prior exposure to raltegravir and multiple integrase resistance mutations.

**Methods:**

We describe two cases of HIV subtype B-infected patients starting dolutegravir after previous failure on a raltegravir-containing regimen with extensive resistance. Genotypic analysis was performed using population sequencing and 454 ultradeep sequencing of integrase at time of raltegravir exposure.

**Results:**

Both patients were diagnosed in early 1990s and received mono- and dual therapy, followed by several cART-regimens. Due to presence of extensive resistance, the genotypic susceptibility score of these regimens never reached a score >2 and never resulted in sustained virological suppression despite good adherence. Early 2012, the clinical condition of patient 1 worsened during persistent failure of a mega-cART regimen despite excellent drug levels. Six major PI, six minor PI, seven NRTI, six NNRTI and two INI mutations plus DM-virus were detected (Table 1). Ultra-deep sequencing of integrase showed the selection of Q148R, E138K+Q148K, and N155H variants and phenotypic raltegravir resistance was demonstrated. After addition of dolutegravir and enfuvirtide to the failing regimen (zidovudine, lamivudine, tenofovir, etravirine, darunavir/ritonavir, maraviroc), viral load (VL) decreased from 244,000 to <20 cps/mL within five months, CD4-count increased (33 to 272 mm^3^) and the clinical condition improved substantially. In patient 2, similar worsening of the clinical condition was observed late 2012 during persistent failure on mega-cART. Five major PI, six minor PI, nine NRTI, seven NNRTI and one INI mutation plus DM-virus were detected. Ultra-deep sequencing showed selection of N155H, followed by Q95K and V151I variants and phenotypic raltegravir resistance was demonstrated. Dolutegravir was added to his failing regimen (zidovudine, lamivudine, etravirine, atazanavir/ritonavir, maraviroc) at a VL of 39,000 cps/mL. Sustained virological suppression was reached within five months with considerable increase of CD4-count (41 to 175 mm^3^) and slight improvement of clinical condition.

**Conclusions:**

We present the first patients with extensive integrase resistance who were treated with dolutegravir in clinical practice and who achieved excellent virological and immunological success. These cases demonstrate the high genetic barrier of dolutegravir.

**Table 1:** Cumulative result of the performed genotypic analyses of patients 1 and 2 before start of dolutegravir and their interpretation by Stanford HIVDB v7.0

	RT	PR	IN
Pt. 1	M41L, D67N, T69i, L74V, M184V, L210W, T215Y, A98G, K103N, V108I, E138G, Y181C, G190A	M46L, I50V, I54V, V82A, I84V, L90M	Q148R, E138K
Pt. 2	M41L, D67d, T69G, K70R, L74I, M184V, L210W, T215Y, K219E, A98G, L100I, K103N, V106I, V108I, E138K, G190A	M46I, I54V, V82C, I84V, L90M	N155H
3TCABCAZTD4TDDIFTCTDFEFVETRNVPRPVATVDRVFPVIDVLPVNFVSQVTPVDTGEVGRAL			
Pt. 1RRRRRRRRRRRRIRRRRRRIRR			
Pt. 1RRRRRRRRIRRRIRRRRRRSRR			

